# ATP-Binding Cassette Subfamily G Member 2 in Acute Myeloid Leukemia: A New Molecular Target?

**DOI:** 10.3390/biomedicines12010111

**Published:** 2024-01-05

**Authors:** Daniela Damiani, Mario Tiribelli

**Affiliations:** 1 Division of Hematology and Stem Cell Transplantation, Udine Hospital, 33100 Udine, Italy; mario.tiribelli@uniud.it; 2 Department of Medicine, Udine University, 33100 Udine, Italy

**Keywords:** acute myeloid leukemia, ABCG2, multidrug resistance, expression regulation, inhibitors

## Abstract

Despite the progress in the knowledge of disease pathogenesis and the identification of many molecular markers as potential targets of new therapies, the cure of acute myeloid leukemia remains challenging. Disease recurrence after an initial response and the development of resistance to old and new therapies account for the poor survival rate and still make allogeneic stem cell transplantation the only curative option. Multidrug resistance (MDR) is a multifactorial phenomenon resulting from host-related characteristics and leukemia factors. Among these, the overexpression of membrane drug transporter proteins belonging to the ABC (ATP-Binding Cassette)-protein superfamily, which diverts drugs from their cellular targets, plays an important role. Moreover, a better understanding of leukemia biology has highlighted that, at least in cancer, ABC protein’s role goes beyond simple drug transport and affects many other cell functions. In this paper, we summarized the current knowledge of ABCG2 (formerly Breast Cancer Resistance Protein, BCRP) in acute myeloid leukemia and discuss the potential ways to overcome its efflux function and to revert its ability to confer stemness to leukemia cells, favoring the persistence of leukemia progenitors in the bone marrow niche and justifying relapse also after therapy intensification with allogeneic stem cell transplantation.

## 1. Introduction

Acute myeloid leukemia is an aggressive disease arising from immature myeloid hematopoietic progenitors that acquire proliferative and survival advantages and defective differentiation capacity, accounting for increased infective and hemorrhagic risk [1]. Standard intensive chemotherapy-based regimens remain central in inducing complete response (CR) in AMLs, with CR rates ranging from 60–85% in younger and 40–60% in older people [2]. Furthermore, relapse occurs early in a high proportion of patients, explaining the disappointing survival rates in adult AML patients and justifying the use of stem cell transplantation as the only curative option for this disease [3]. The recent advances in diagnostic tools significantly increased the knowledge of leukemia pathogenesis and permitted the development of prognostic risk scores based on the different genetic/karyotypic alterations and the identification of many molecular markers as potential targets for new therapies [4]. Moreover, the genetic-based risk classification highlighted, once again, the prognostic importance of the initial response to chemotherapy and of the persistence of minimal residual disease [5]. In acute myeloid leukemia, as well as in many other cancer types, a recognized role in affecting the initial response to chemotherapy has been attributed to the efflux pump activity of the tree most studied members of the ABC protein family, ABCB1, ABCC1, and ABCG2. By diverting transported drugs from their intracellular target, they reduce therapy efficacy and induce the Multidrug Resistance (MDR) phenomenon, by which neoplastic cells acquire cross-resistance to different “conventional” structurally unrelated anticancer molecules, still representing the backbone of cancer treatment [6,7,8,9], but also restrict the activity of many new molecular target drugs [10]. However, more recent studies suggest that ABC transporters may have many other important roles in cancer cell biology [11], despite a mechanistic understanding of how these functions contribute to tumor biology that is still lacking. In experimental models, there is a growing body of evidence that the loss or inhibition of various ABC transporters can influence tumor cell phenotype, proliferation, differentiation, migration, and survival [12,13,14], as well as tumorigenesis and tumor progression [12,15,16]. On this basis, counteracting ABC transporter activity may have additional benefits beyond efflux inhibition and could be effective regardless of the type of drugs employed. In this paper, we focused on ABCG2, one of the most recently identified components of the ABC superfamily, revising the current knowledge on its expression and prognostic role in acute myeloid leukemia and on the possible methods for reverting its activity.

## 2. ABCG2: Nomenclature, Structure, and Physiological Function

The human genome contains 49 genes that encode ABC proteins, organized into seven subfamilies (ABCA–ABCG) [17]. They have many physiological functions by exporting xenobiotics, proteins, and metabolic products in tissues [18], and they can export a myriad of compounds with unrelated chemical structures, decreasing their intracellular accumulation and, as a consequence, their therapeutic efficacy [19]. Among them, ABCG2 was discovered in the late 1990s by Doyle et al. in the MCF-7 drug-resistant breast cancer cell subline and, for this reason, initially named Breast Cancer Resistance Protein (BCRP) [20]. In the same year, Allikmets et al. cloned the DNA of an ABC protein involved in multidrug resistance specific to the human placenta (human placenta-specific ATP binding cassette protein, ABCP) [21]. In 1999, Miyake et al. first cloned the DNA of a protein with high homology to ABC proteins from mitoxantrone-resistant colon carcinoma cells, justifying the name of the mitoxantrone resistance protein (MXR protein) [22]. As the second discovered member of the ABCG subfamily, the protein was further designed as ABCG2 by the Human Genome Organization Committee and assigned to the 338 cluster of differentiation (CD338) of the antigen nomenclature system by the Human Cell Differentiation Molecules organization. The human gene encoding for ABCG2 is located on chromosome 4q22 and spans over 60 kilobases, containing 16 exons and 15 introns. The translational start site is in exon 2, the ATP binding sites are in exons 3 and 6, and the ABC signature motif is in exon 6 [23,24]. The mechanisms controlling *ABCG2* expression are not well clarified, but in cell lines with high ABCG2 levels, multiple rearrangements on chromosome 4, including balanced translocations between chromosomes 4 and 7, have been found [25]. Moreover, many factors are known to regulate *ABCG2* at the transcriptional, splicing, and epigenetic levels [24]. Transforming growth factor beta, interleukin 1, and tumor necrosis factor-alpha seem to decrease mRNA and ABCG2 protein [26,27], and insulin-like growth factor 1 increases protein expression [27]. ABCG2 is a 72-kDa protein composed of 665 amino acids. Unlike ABCB1 and ABCC1, ABCG2 is considered a half-transporter with only one nucleotide-binding domain (NBD) and one transmembrane domain (TMD). The minimal functional unit of ABCG2 is a homodimer, but the presence of higher-order oligomers is also possible [28,29], in contrast to other ABCG members, such as ABCG5 and ABCG8, whose functional form results from heterodimerization [30]. ABCG2 dimerization occurs in the endoplasmic reticulum (ER), then ABCG2 moves to the Golgi apparatus, where it undergoes further post-translational quality control before being transferred to the apical membrane of the cell [31]. ABCG2 undergoes complex post-transcriptional controls in the ER that have a crucial role in membrane protein topogenesis, facilitating its insertion, orientation, and folding and, ultimately, attaining its quaternary structure. In these mechanisms, many chaperon proteins, such as the binding immunoglobulin protein (BiP) or calnexin/calreticulin (CALN/CALR), are involved. Moreover, ER is involved in quality control of the newly synthetized proteins, which can be transferred to the Golgi apparatus only in correctly folded conformation. Two additional quality control steps are undergone in the Golgi apparatus and in the cell periphery, including internalization, recycling, and lysosomal degradation, all of which are potential targets for ABCG2 therapeutic modulation. For a detailed description of ABCG2 synthesis, see Sarkadi et al. [32].

**Figure 1:**
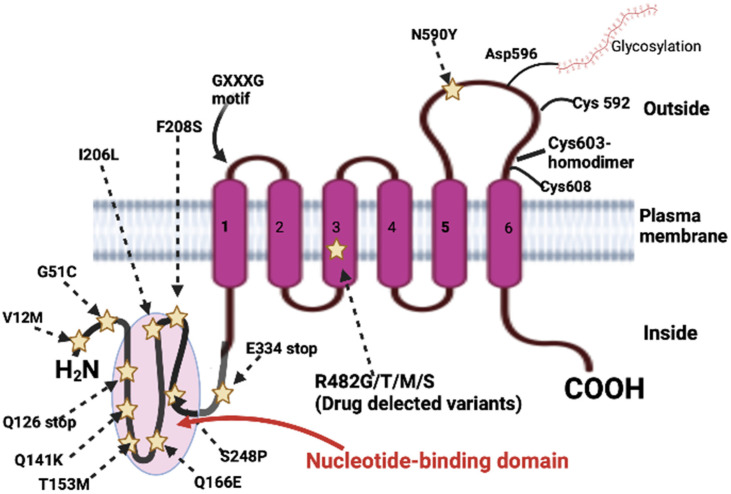
ABCG2 structure and variants at the N-terminus nucleotide-binding domain (NBD) in purple transmembrane domains (TMDs) [Adapted from Noguchi et al. [36]].

Compared to most other ABC transporters on the cell membrane, ABCG2 is in a reverse configuration, with an N-terminal ATP binding site and a C-terminal transmembrane domain [24]. The transmembrane domain of ABCG2 (residues 361 to 655) has six transmembrane segments and an extracellular loop between segments five and six. Glycosylation is present only at asparaginase 596 in the extracellular loop, but this does not impact proper protein localization or function [33]. The GXXXG sequence located in transmembrane domain 1 plays a role in dimerization and may also be important in the formation of higher-order complexes [34]. Cysteine 603 in the extracellular loop is involved in the formation of intramolecular disulfide bonds and can contribute to dimerization. The intramolecular disulfide bonds between cysteine 592 and cysteine 608 affect its function and stability [35]. A schematic representation of the membrane organization and residues or mutations affecting the transport function is shown in Figure 1.

Although ABCG2 was first discovered in multidrug-resistant cell lines, it was soon demonstrated in normal tissues, where it is believed to be involved in protecting tissues from xenobiotic accumulation and toxicity. ABCG2 is located on the apical surface of epithelial cells [37], and a physiologically high level of proteins has been demonstrated in strategic sites such as the central nervous system, liver, adrenal gland, placenta, hematopoietic stem cells, breast, small intestine, and colon [38,39], supporting its role in tissue defense. Like ABCB1 and ABCC1, ABCG2 confers resistance to many amphipathic natural anticancer drugs and methotrexate. In addition to natural product-type drugs, ABCG2 reduces the accumulation of porphyrins, precursors of the heme biosynthetic pathway within cells [40], and it has been suggested that ABCG2 protects stem cell survival by reducing heme accumulation under hypoxic conditions [40]. Besides the efflux of natural anticancer drugs, ABCG2 is involved in the excretion of physiological metabolites, in preventing or limiting intestinal absorption, and in enhancing biliary efflux of dietary carcinogens, such as 2-amino-1-methyl-6-pheylimidazo[4,5-b] pyridine (PhIP), the most important heterocyclic amine formed from frying and cooking meat [41]. 

## 3. ABCG2 Substrates

As ABCG2 was demonstrated in multidrug-resistant cancer cell lines, the first identified protein substrates were chemotherapeutic agents such as mitoxantrone, flavopiridol, methotrexate, irinotecan, and its active metabolite SN-38. Since then, many other classes of substrates have been identified, and the list is still rapidly expanding, including antivirals, antibiotics, antiHMG-CoA reductase inhibitors, calcium channel blockers, carcinogens, and tyrosine kinase inhibitors. In general, the compounds transported by ABCG2 are large, hydrophobic molecules that are positively or negatively charged. A list of selected ABCG2 substrates is shown in Table 1.

The precise mechanism of substrate transport is still not well understood. In all ABC proteins, ATP is bound by the Walker A and B motifs of one NBD and by the signature sequence of the other NBD, resulting in a so-called “sandwich dimer” due to a close interaction of the two NBDs [79]. Recent publications have elucidated the molecular mechanism of action of ABCG2. According to data provided by cryogenic electron microscopy, the transport is linked to conformational changes of the TMDs, which are regulated by the ATP-dependent formation and separation of the NBD “sandwich dimer.” Compared to ABCB1, conformational changes in ABCG2 during the transport cycle are less large, as the NBDs are already in close proximity in the nucleotide-free state [80]. In the absence of ATP, ABCG2 adopts an inward-facing (IF) conformation, with the transmembrane cavity open toward the cytosolic side of the cell membrane and the central four transmembrane helices (TH2, TH5, TH2′, TH5′) arranged to form a large hydrophobic slit-like drug-binding pocket (cavity1). When ATP is bound, ABCG2 is set in the outward-facing (OF) state, and the substrate is forced into the smaller and less hydrophobic cavity 2, allowing the release of the substrate to the extracellular side. The change in substrate affinity in the IF and OF conformations favors substrate uptake on the cytosolic side and its release on the extracellular side [81,82].

## 4. ABCG2 in Acute Myeloid Leukemia: Expression and Prognostic Role

Despite the heterogeneity of employed methods (m-RNA quantification, protein expression by flow cytometry or immune-cytochemistry, protein function by efflux of naturally fluorescent substrates) and the lack of standardization that makes data comparison difficult, many studies in the past decades have reported a relationship between ABCG2 overexpression and poor clinical outcomes in AML. 

A comprehensive review of ABCG2 expression and clinical impact in AML has been recently published [83].

In summary, most data support the negative role of ABCG2 on AML outcomes, both in adults and in children. Interestingly, its negative impact also emerges when evaluating *ABCG2* transcription despite the known high post-transcriptional manipulation of the protein. The frequent ABCG2 and ABCB1 co-expression on leukemia cells, with synergistic effects on drug efflux, may have a role in reducing the complete remission rate and in favoring early relapse. However, it must be considered that ABCG2 overexpression may also contribute to the negative prognosis by conferring stem cell-like properties to leukemia cells, thus favoring the persistence of disease in the hypoxic bone marrow niche and accounting for “late” relapses occurring after chemotherapy intensification and stem cell transplantation. 

## 5. ABCG2 Reversal: Counteracting Efflux Activity

With this premise, the identification of strategies able to overcome multidrug resistance appears crucial to improving the leukemia cure and patients’ survival. Over the past three decades, many strategies, e.g., the synthesis of drugs eluding ABC transporters or the use of drug combinations to take advantage of the “collateral sensitivity” phenomenon, have been proposed with disappointing results. The most pursued way was to prevent multidrug resistance by inhibiting ABC protein efflux activity and re-sensitizing resistant cells to conventional chemotherapy. Most of the studies tried a combination therapy aimed at inhibiting transporter function by inhibitor molecules and then induced leukemia cell death by using chemotherapeutic drugs. These combined therapies, initially used to inhibit ABCB1, demonstrated the best results in vitro [84,85] and displayed some encouraging results in vivo, limited by off-target toxicity. So far, there are no approved sensitizers for clinical use. Among molecules able to inhibit ABCG2-mediated efflux, the first identified was fumitremorgin C (FTC), a mycotoxin produced by Aspergillus fumigatus. Since then, many other potent ABCG2 inhibitors have been identified by random screening of different classes of drugs sharing structural characteristics known to favor ABCG2 inhibition [86] or by drug repurposing, which has the advantage of shortening the drug approval process [87] and can take advantage of known pharmacokinetic indexes [87]. Among them, the most interesting are tivozanib, fostamatinib, ponatinib, and febuxostat, all active at nanomolar IC_50_ and compatible with clinical use [88,89,90]. Some inhibitors selectively inhibit ABCG2 efflux. Others were first selected as ABCB1 blockers and also demonstrated anti-ABCG2 activity [91,92]. A few compounds, such as vandetanib, showed dual ABCG2 and ABCC1 inhibition [93], but at present, only a major curcumin metabolite, tetrahydrocurcumin, seems to act as a pan-inhibitor (anti-ABCB1,-ABCC1,-ABCG2) [94]. The advantage of multi-inhibitors on leukemic cells is evident, considering the frequent co-expression of ABC proteins, but the cumulative off-target toxicity remains an unsolved problem. At present, over one hundred molecules distributed in 45 classes of compounds have shown in vitro inhibitory activity toward ABCG2; many of them demonstrated inhibitory effects only at high concentrations, not reachable in vivo. However, it must be underlined that in vitro tests were performed in systems with very high ABCG2 expression and that, in wild leukemia samples, ABCG2 levels are significantly lower than in cell lines, and ABCG2 inhibition may be reached with lower concentrations. Table 2 lists some ABCG2 inhibitors by class of compound selected through in vitro tests, with IC_50_ attainable in clinical settings. 

Recently, other ABCG2 inhibitors have been identified by drug repurposing. Ribociclib, a CDK4/6 inhibitor approved for the treatment of locally advanced/ metastatic breast cancer, revealed ABCB1 and ABCG2-mediated daunorubicin and mitoxantrone efflux inhibition in AML cell lines and in CD4+/Flt3-WT leukemia cells [117]. In vitro dual inhibition of ABCC1 and ABCG2-mediated daunorubicin and mitoxantrone efflux was demonstrated by talazoparib, a TK inhibitor used in metastatic BCRA1/2-mutated breast cancer [118]. Tucatinib, a selective HER-2 inhibitor, approved in 2020 for the treatment of HER-2+ breast cancer, showed high ABCG2 inhibition activity in primary leukemia blast cells and in the HL60/ABCG2 cell line, increasing cell death, Hoechst 3342 cell accumulation, and significantly reducing the leukemia stem cell population [119]. ABCG2-mediated efflux block was also demonstrated by venetoclax, bcl-2 inhibitors originally approved for lymphoproliferative diseases and recently employed in elderly acute myeloid leukemia in association with hypomethylating agents [120]. In vitro and modeling studies have demonstrated high ABCG2 inhibition by bexarotene and by vemurafenib, a BRAF inhibitor approved for melanoma and used in the treatment of hairy cell leukemia [121]. Cermáková et al. reported that zanubrutinib, a second-generation Bruton tyrosine kinase inhibitor approved for relapsed/refractory marginal zone lymphoma (MZL), mantle cell lymphoma (MCL), and Waldenström’s macroglobulinemia (WM), counteracts anthracycline resistance by targeting aldo-keto reductase 1C3 (AKR13) and inhibits daunorubicin efflux mediated by ABCB1, ABCC1, and ABCG2, suggesting a possible use in acute myeloid leukemia [122].

The recent advances in the knowledge of ABCG2 structure and transport mechanism will be helpful in discovering new potential inhibitors, modifying known molecules, or starting from new scaffolds. It is well recognized that inhibition potency is closely related to drug physicochemical properties, like hydrophilic/lipophilic balance, which reflect solubility and permeability on biological membranes [123]. Lipinski et al. proposed “the rule of 5” regarding solubility and permeability to guide new drug development, considering that for ABCG2 inhibition, solubility is necessary for membrane penetration, and only hydrophobic moieties can bind TMDs [124]. Moreover, the “ideal” ABCG2 inhibitor should combine high potency of inhibition, low/null toxicity, and high ABCG2 selectivity. Moinul et al. investigated the structure-inhibition relationship of many molecular scaffolds, analyzing their different pharmacological activities, and proposed the minimum possible structural features required for the design of new ABCG2 inhibitors. They identified a benzene ring fused with a heterocyclic aromatic ring as essential for activity and suggested many additional groups to modulate polarity to improve TMD binding and selectivity [125].

## 6. ABCG2 Reversal: Affecting Protein Expression

In the past years, the study of ABCG2 activity highlighted that beyond drug efflux, it contributes to changing leukemia cell biology, favoring the acquisition of stem cell properties, resulting in protection from drug toxicity and immune surveillance, and eventually preventing disease eradication also after stem cell transplantation. Moreover, the study of complex molecular pathways involved in ABCG2 expression has underlined that many of them are also involved in cancer/leukemia pathogenesis.

### 6.1. Regulation by Transcription Factors

To date, several cis-regulatory elements and transcription factor binding sites in the ABCG2 gene region have been identified, many of them expressed in a tissue-specific manner, many of them overexpressed in acute myeloid leukemia, and often already targets of new anticancer molecules. 

Krishnamurthy et al. found that ABCG2 transcription is activated by the binding of hypoxia-inducible factor 1α (HIF1α)/ARNT heterodimer to a hypoxia response element (HRE) under low oxygen conditions [40], enhancing hypoxic cell survival, protecting hematopoietic stem cells in the bone marrow niche, and also leukemia stem cells. High levels of HIF1α were reported in acute myeloid leukemia in de novo AML patients by Jabari et al. [126]. Xu et al. observed a positive correlation between HIF1α and ABCG2 expression [127], suggesting that HIF1α could be considered a prognostic indicator in AMLs and making HIF1α a potential therapeutic target to down-modulate ABCG2 expression and eliminate leukemia stem cells [128]. 

*C-Myc* is a proto-oncogene and encodes an HLH-leucine zipper transcription factor to regulate gene expression [129]. Overexpression of c-MYC in human mammary epithelial cells results in enhanced ABCG2 expression and activity. In the leukemia setting, high expression of c-MYC-driven ABCG2 was observed in CD34 cells of chronic myeloid leukemia [130]. However, Zhang et al. demonstrated that c-MYC can access the ABCG2 promoter only if its binding sites are unmethylated [131]. In FLT3-ITD acute myeloid leukemia, the combination of homoharringtonine and quizartinib AKT and its downstream targets, including c-MYC, reduces the ABCG2 overexpressing side population [132].

As c-MYC, also one other HLH transcription factor, E2F1 is often overexpressed in many cancers and acts by directly bonding the ABCG2 promoter, activating protein transcription, and inducing chemotherapy resistance [133]. In vitro studies in leukemia cell lines K562 and K562/A02 transiently transfected with miR-98 show that miR-98 upregulation results in reduced E2F1 levels, a reduction of ABCG2 levels, and increased chemosensitivity [134].

Nuclear factor kappa B (NF-kB) consists of five subunits that form homo or hetero dimers to bind a gene promoter [135]. In acute leukemia, the activation of the NF-kB pathway affects cell survival, promotes immune evasion, favors proliferation, induces drug resistance, and enhances the stem cell population [136]. Studies in cancer cell lines prove that NF-kB directly binds the ABCG2 promoter, enhancing BCG2 expression and membrane translocation of the protein [137].

Spalt-like transcription factor 4 (SALL4) is one of the transcription factors involved in maintaining the pluripotency of stem cells. In acute myeloid leukemia, Jeong et al. demonstrated its aberrant overexpression in myeloid leukemia and a correlation between SALL4 and ABCG2 levels [138]. Moreover, they showed that SALL4 modulates ABCG2, not the binding promoter but some distal region, or represses PTEN expression [139]. 

Szatmari et al. demonstrated ABCG2 upregulation via activation of peroxisome proliferator-activated receptor (PPAR)-γ in human myeloid dendritic cells [140]. Recently, Kim et al. demonstrated high ABCG2 expression levels in M2 macrophages [141]. They reported that IL-4 and IL-13, responsible for macrophage polarization to M2 type, induce PPARγ transcription, PPARγ bound to the ABCG2 promoter, and ultimately ABCG2 overexpression in M2 macrophages, limiting the intracellular accumulation of macrophage-targeting antibiotics [141]. In acute myeloid leukemia, PPARγ stimulation exerts an opposite regulatory effect. It results in phosphatase and tensin homolog (PTEN) upregulation and consequent ABCG2 internalization through reduced Akt phosphorylation [142,143].

Nrf2, a nuclear factor-erythroid 2-related transcription factor, plays a critical role in the transcriptional regulation of many metabolizing enzymes that rescue cells from oxidative stress and is found to be a transcription factor for ABCG2 [144]. In acute myeloid leukemia, overexpression of Nrf2 was observed at diagnosis and relapse [145] and has been associated with disease progression and increased resistance to cytarabine in cases with poor prognostic gene mutations [146]. Hu et al. hypothesized that Nrf2-mediated drug resistance occurs via JNK/NF-kB signaling pathway activation, both involved in the regulation of ABCG2, which may contribute to chemotherapy resistance [147]. In pancreatic cancer cell lines, knockout Nrf2 re-sensitized cancer cells to 5-Fluorouracil by suppressing ABCG2 expression [148]. Moreover, Choi et al. reported that Nrf2 increased ABCG2 expression by modulating the microRNA miR-206 level [149].

### 6.2. Regulation by Kinase Signaling

Many kinases, such as PIM1, PI3K/AKT, ERK, and JNK, are involved in ABCG2 expression, activity, and trafficking [150], either by direct phosphorylation of ABCG2 or indirectly by phosphorylation of downstream effector kinases/regulatory proteins. Among them, PIM1 kinase phosphorylates ABCG2 at threonine 362, promotes protein membrane translocation, stabilization, and multimerization, and enhances its activity. PIM1 inhibitors display a dual-mode effect on ABCG2-expressing cells. They impair efflux function and reduce protein levels on the membrane [151]. The PI3K/AKT pathway also mediates ABCG2 sequestration in extracellular micro-vesicles (ESs), contributing to the dissemination of drug resistance [152]. Pharmacologically inhibition of the PI3K/AKT pathway transfers ABCG2 from ESs to the cell compartment, reversing drug resistance [152]. In addition, PI3K/AKT mediates ABCG2 at the transcriptional level [153]. JNK pathways and consequent upregulation of ABCG2 are activated by high levels of CXCR4, a chemokine receptor frequently expressed in AML [154]. Finally, Wnt and Hedgehog pathways, involved in the maintenance of leukemia stem cell survival, bind the ABCG2 promoter and increase protein expression [155].

### 6.3. Epigenetic Regulation

Epigenetic changes commonly involve DNA methylation and/or post-translational modification of histone proteins, with permissive or repressive effects on gene expression. Hypermethylation of the CpG island of the *ABCG2* promoter by DNMT1 and DNMT3a and recruitment to the methylated site of methyl CpG binding domain proteins (MBDs) along with co-repressors mSIN3a and HDAC1 lead to gene silencing [156]. This is associated with HDAC1-mediated deacetylation at H3K9 and with histone methyltransferases’ (HMTs) addition of the repressive histone mark H3K9me3 [156]. ABCG2 upregulation has been observed in cell lines after treatment with 5-aza-2′ deoxycytidine (5-AZA) [157,158]. Given the large clinical use of 5AZA in older patients with AML, the possible impact on clinical outcomes deserves further investigation. Histone modification by acetylation, methylation, phosphorylation, citrullination, or ubiquitination of lysine and arginine can result in enhanced gene expression or suppression. ABCG2 transcriptional activation results from the replacement of the repressive histone mark H3K9me3 with permissive changes, including H3K4me and H3S10p, the release of HDCA1s, and the recruitment of chromatin remodeler Brg-1 and RNA polymerase II to the promoter. Loss of function mutant of enhancer of zeste homologue 2 (EZH2), an enzymatic component of polycomb repressive complex 2 (PRC2), increases ABCG2 mRNA expression and contributes to myelodysplatic syndrome pathogenesis [159]. Histone deacetylase inhibitors, such as romidepsin, vorinostat, and panobinostat, have demonstrated the ability to increase ABCG2 synthesis and function [160]. Developing drugs able to inhibit histone acetyltransferase or activate histone deacetylase may be a new strategy for decreasing ABCG2 expression. Moreover, post-transcriptional regulation of ABCG2, with increased mRNA stability, is the product of the bound to ABCG2 mRNA of the hypermethylated RNA recognition motif (RRM) of heterogeneous nuclear ribonucleoprotein A1 (HnRNPA1) [161].

MicroRNAs (miRNAs) are small, non-coding RNAs able to cause mRNA degradation and/or decreased translational efficiency, affecting protein expression and functions [162]. Several studies have shown that miRNAs regulate ABCG2 mRNA or protein expression. MiR-487a, mi-R-328, mi-R-181a, and mi-R-302 were demonstrated to increase mitoxantrone sensitivity in cell lines and reduce ABCG2 expression in cell lines [163,164,165,166]. Mi-R-519c may be involved in ABCG2 overexpression, affecting mRNA stability [156].

### 6.4. Regulation by Protein Degradation

At least three degradation pathways have been identified through “in vitro” studies on cell lines. The first mechanism involves Derlin1-mediated block of endoplasmic reticulum (ER) to Golgi transport that affects protein maturation [167]. Moreover, Derlin-1 facilitates ubiquitin-mediated ER-associated degradation (ERAD) of both glycosylated and non-glycosylated forms [167]. In cancer cell lines, this mechanism was induced by low molecular weight heparin (LMWH). The second mechanism is endocytosis-mediated internalization and lysosomal degradation of ABCG2. The functional consequence of protein down-regulation has been demonstrated by the increased sensitivity of mitoxantrone in the MCF7-MX cell line [168]. Wei et al. demonstrated that sorafenib, a multi-kinase inhibitor employed in hepatocarcinoma and in relapsed FLT3-ITD acute myeloid leukemia, not only inhibits ABCG2 efflux activity but induces ABCG2 degradation in the lysosome [169]. The third mechanism is ABCG2 half-life reduction through calpain-mediated protein degradation. This mechanism was described by Hu et al., who tested the cytotoxic effect of Escallonia acid D (SAD), a toxic metabolite of Penicillium oxalicum, in the side population of a lung cancer cell line. They observed that SAD treatments reduced side population size by reducing membrane ABCG2 and demonstrated that protein down-modulation was due to the acceleration of ABCG2 degradation by activation of the calpain pathway [170].

On this basis, a new approach to counteract ABCG2 may be hypothesized, targeting the protein rather than its function. A partial selection of molecules with demonstrated activity on ABCG2 expression in cancer cell lines is reported in Table 3. 

## 7. Conclusions

Since its first description in the late 1990s, several studies have associated ABCG2 overexpression with poor outcomes in acute myeloid leukemia. Despite this, very few efforts have been made in the attempt to counteract its function, probably as a consequence of the disappointing results in the clinical use of ABCB1 inhibitors, almost due to their off-target toxicity. However, the recent explosion of anti-cancer target drugs, often substrates of ABCG2, has led to the identification of several compounds with high ABCG2 affinity and strong efflux inhibition ability that may be “repurposed” as ABCG2 reversal agents. Moreover, knowledge of the chemical structure of molecules with proven inhibitory activity helps better understand the mechanisms of ABCG2-mediated transmembrane transport and paves the way for the development of new molecules combining high inhibition capacity with reduced toxicity. In addition, the discovery of mechanisms underlying leukemia pathogenesis has highlighted that many of them are involved in ABCG2 regulation, so identifying potential therapeutic targets to indirectly inhibit ABCG2 expression and offering a new perspective to counteract ABCG2-mediated drug resistance is able to modify leukemia cell biology. At present, data on drugs targeting ABCG2 transcription, internalization, and stability are quite scarce or preliminary but clearly warranted, along with extensive studies on cellular pathways specifically activated in leukemia cells and efforts toward the standardization of methods to evaluate protein expression and function.

## Figures and Tables

**Table 1:** ABCG2 substrates by class of compounds.

**Chemotherapy agents**
Mitoxantrone [22], Daunorubicin [42], Doxorubicin [42], Epirubicin [42], Idarubicin [42], Bisantrene [42], Flavopiridol [43], Etoposide [44], Teniposide [44], Topotecan [45] Irinotecan [46], Diflomotecan [47], Homocamptothecan (weak) [47], Methotrexate [48], Tomudex [49], Imatinib [50], Nilotinib [51], Gefitinib [52], Canertinib [53]Triazolacridones [54]
**Antivirals**
Zidovudine [55], Lamivudine [56], Abacavir [57]
**HMG-CoA reductase inhibitors**
Rosuvastatin [58], Pitavastatin [59], Cerivastatin [60]
**Porphyrins**
Pheophorbide A [61], Chlorin e6 [61], Protoporphyrin IX [61], Phytoporphyrin [62]
**Flavonoids**
Genistein [63], quercetin [64]
**Antibiotics**
Ciprofloxacin [65], Ofloxacin [65], Norfloxacin [65], Erythromycin [66], Rifampicin [66], Nitrofurantoin [67], Sulfasalazine [68]
**Benzimidazoles**
Albedazole sulfoxide [69], Oxfendazole [69], Pantoprazole [70]
**Pyridines**
Nitrendipine [71], Azidopine [71], Dipyridamole [72], Folic acid [73], Cimetidine [74], Riboflavin [75]
**Fluorescent compounds**
Hoechst 33342 [76], Rhodamin 123 [77], Lysotracker green [77], BODIPY-prazosin [77]
**Carcinogens**
Aflatoxin B [78], 2-amino-3-methylimidazo[4,5-f]quinoline [78], 3-amino-1,4-dimethyl-5H-pyrido[4,3-b]indole [78], 2-amino-1-methyl-6-phenylimidazo[4,5-b]pyridine [74]

**Table 2:** ABCG2 inhibitors are listed by structural class.

Structural Class	Compound	IC50 (μM)	Ref.
Chalcones	Indolylphenylproenone	0.27	[95]
Chromones	Chromone4a	0.086	[96]
	Chromone31	0.046	[97]
Diketopiperazines	Ko143(FTC analog)	0.01	[98]
Flavonoids	Flavone	2.8	[99]
	6-prenylchrysin	0.29	[100]
	Flavonoid dimer	1	[99]
Hedgehog pathway inhibitors	Vismodegib	1.4	[101]
Isocitrate dehydrogenase inhibitors	Enasidenib	1	[102]
Immunosuppressants	Sirolimus	1.9	[103]
Non-purine xanthine oxidase inhibitors	Febuxostat	0.027	[90]
	Topiroxostat	0.18	[90]
	Benzbromarone	0.2	[90]
ABCB1 inhibitors	Tariquidar	0.9	[104]
	Tariquidar derivative 6	0.06	[104]
Indenoindole-type derivatives	Indeno[1,2-b] indole	0.21	[105]
	9-hydroxyindeno[1,2-b] indole	0.21	[106]
	Indeno[1,2-b] indole homodimer	0.024	[107]
Tariquidar-related triazoles	IR-ST1	0.14	[108]
	UR-MB108	0.079	[108]
Thrombopoietin receptor	Eltrombopag	3.1	[109]
Tyrosine kinase inhibitors	Alectinib	1.5	[110]
	Bosutinib	2	[111]
	Dasatinib	2	[111]
	Erlotinib	0.13	[36]
	Fostamatinib	0.05	[112]
	Gefitinb	0.5	[113]
	Ponatinib	0.04	[89]
	Vandetanib	0.2	[93]
	Tivozanib	0.07	[88]
	Imatinib	1	[114]
	Ulixertinib	1	[115]
	Dacomitinib	1	[116]

**Table 3:** Modulators of ABCG2 expression by regulation site.

Regulation Level	Drug	Ref.
Transcription	Vorinostat	[171]
	Trichostatin A	[171]
	Fasudil (rho kinase inhibitor)	[172]
	Sulbactam	[173]
	Ly294002 (PIK3 inhibitor)	[174]
	SP600125 (JNK inhibitor)	[175]
	Resveratrol	[176]
	Fucoxanthin	[177]
	Genistein	[178]
	Vinblastine	[179]
	Gefitinib	[174]
	Telatinib	[180]
	Glasdegib	[181]
	Toremifene	[182]
	Dexamethasone	[183]
Protein degradation	Sorafenib	[169]
	PD98059 (MEK inhibitor)	[184]
	UO126 (MEK inhibitor)	[184]
	Imatinib	[185]
Protein internalization	Ly294002 (PIK3 inhibitor)	[186]
	Fasudil	[172]
	Rapamycin	[187]
	NVP-BEZ235 (dualPI3K/mTOR Inhibitor)	[188]
	Pioglitazone	[142]

## Data Availability

No new data were created or analyzed in this study. Data sharing is not applicable to this article.
